# Lectins and lectibodies: potential promising antiviral agents

**DOI:** 10.1186/s11658-022-00338-4

**Published:** 2022-05-13

**Authors:** Mohsen Nabi-Afjadi, Morteza Heydari, Hamidreza Zalpoor, Ibrahim Arman, Arezoo Sadoughi, Parisa Sahami, Safiyeh Aghazadeh

**Affiliations:** 1 Department of Biochemistry, Faculty of Biological Science, Tarbiat Modares University, Tehran, Iran; 2 Institute of Biochemistry and Biophysics, University of Tehran, Tehran, 13145-1384 Iran; 3 Shiraz Neuroscience Research Center, Shiraz University of Medical Sciences, Shiraz, Iran; 4 Network of Immunity in Infection, Malignancy and Autoimmunity (NIIMA), Universal Scientific Education and Research Network (USERN), Tehran, Iran; 5 American Association of Kidney Patients, Tampa, FL USA; 6 Department of Molecular Biology and Genetics, Faculty of Sciences and Arts, Zonguldak Bulent Ecevit University, Zonguldak, Turkey; 7 Department of Immunology, International Campus, Shahid Sadoughi University of Medical Sciences, Yazd, Iran; 8 Medical Biology Research Center, Health Technologies Institute, Kermanshah University of Medical Sciences (KUMS), Kermanshah, Iran; 9 Division of Biochemistry, Department of Basic Sciences, Faculty of Veterinary Medicine, Urmia University, Urmia, 5756151818 Iran

**Keywords:** Lectins, Lectibody, Carbohydrates, Virus envelope, SARS-CoV-2, HIV, EBV, HCV

## Abstract

In nature, lectins are widely dispersed proteins that selectively recognize and bind to carbohydrates and glycoconjugates via reversible bonds at specific binding sites. Many viral diseases have been treated with lectins due to their wide range of structures, specificity for carbohydrates, and ability to bind carbohydrates. Through hemagglutination assays, these proteins can be detected interacting with various carbohydrates on the surface of cells and viral envelopes. This review discusses the most robust lectins and their rationally engineered versions, such as lectibodies, as antiviral proteins. Fusion of lectin and antibody’s crystallizable fragment (Fc) of immunoglobulin G (IgG) produces a molecule called a “lectibody” that can act as a carbohydrate-targeting antibody. Lectibodies can not only bind to the surface glycoproteins via their lectins and neutralize and clear viruses or infected cells by viruses but also perform Fc-mediated antibody effector functions. These functions include complement-dependent cytotoxicity (CDC), antibody-dependent cell-mediated cytotoxicity (ADCC), and antibody-dependent cell-mediated phagocytosis (ADCP). In addition to entering host cells, the severe acute respiratory syndrome coronavirus 2 (SARS-CoV-2) spike protein S1 binds to angiotensin-converting enzyme 2 (ACE2) and downregulates it and type I interferons in a way that may lead to lung disease. The SARS-CoV-2 spike protein S1 and human immunodeficiency virus (HIV) envelope are heavily glycosylated, which could make them a major target for developing vaccines, diagnostic tests, and therapeutic drugs. Lectibodies can lead to neutralization and clearance of viruses and cells infected by viruses by binding to glycans located on the envelope surface (e.g., the heavily glycosylated SARS-CoV-2 spike protein).

## Introduction

As proteins without any enzymatic activity, lectins are ubiquitous among various kinds of living things. These proteins act as adhesion molecules in the colonization processes of bacteria, archaea, protists, and fungi. Also, they play an essential role in the defense mechanisms and nodulation of Plantae. Interestingly, they are involved in various functions of Animalia, such as cell migration and adhesion, opsonization, immune responses and phagocytosis, and glycoprotein production. These proteins act as cell-agglutinating and carbohydrate-specific proteins and recognition molecules in cell–molecule and cell–cell interactions. Regarding these functions of lectins, they play an indispensable role in many biological processes and mediate biological recognition events. Therefore, they can be a suitable tool for investigating carbohydrate indicators on cell surfaces, mainly the modifications occurring in malignancies and isolation and characterization of glycoproteins.

Infection with enveloped viruses (e.g., coronaviruses and HIV) is one of the leading causes of mortality and morbidity globally, even with the recent development of highly effective direct-acting antivirals. These viruses have envelope glycoproteins that are heavily glycosylated with a high proportion of high-mannose-type glycans (HMGs), protecting them from antibody neutralization and enabling them to interact with cell entry receptors. However, there is no approved therapeutic targeting this potentially druggable biomarker.

HMGs are highly rare in eukaryotic cell-surface glycoproteins because of the processing steps conducted by the Golgi apparatus that make them hybrid and complex glycoforms. Interestingly, despite eukaryotic cells, HMGs are plentiful at the surface glycoproteins of various viruses, shielding them against attacks from the host immune system. Therefore, glycosylation is pivotal for a broad range of functions in viruses: entrance into host cells; protein expression and assembly, and evading the immune system, for example, are the significant contributions of glycosylation of virus surface proteins. Hence, lectins as agents that identify and neutralize virus-associated glycans, particularly HMGs, are valuable tools in antiviral medicine as potent antiviral microbiocides. The antiviral lectins that have been used against human viral infections are from two origins, endogeneous or exogeneous (endo- and exolectins, respectively), the former being animal lectins expressed in a variety of cell types and carrying out a diverse range of activities such as the first-line defense in innate immune system, mediation of cellular adhesion, regulation of glycoprotein synthesis, and signal transduction, while the latter (the focus of this article) are lectins originating from other species. All the exolectins assessed herein have the typical, protein nature, except pradimicin A (PRM-A), which is an antibiotic with a nonprotein origin [1–4].

Although lectin’s attachment to monosaccharides is weak, in the case of more complex ligands, they employ subsite and subunit multivalency to improve the affinity and specificity of their interactions. Bivalent or multivalent lectins reversibly and selectively interconnect with their ligands (carbohydrates or glycoproteins) in solution or on the cell surface. Therefore, this class of proteins is detectable through agglutination assays. In this regard, plant lectins, because of their high stability even in unfadable pH/temperature and exposure to insect/animal proteases, are widely used in clinical and experimental biology and medicine, given their abundant availability; For example, serology arrays were the first field of medicine to use lectins, to distinguish human blood types based on their carbohydrate indicators [1–3, 5].

Accordingly, the diversity of lectins and their capacity to bind to carbohydrates have led to their widespread application in several fields of sciences, including biochemistry, cellular and molecular biology, immunology, pharmacology, medicine, and clinical analysis. Hence, in addition to their antiviral effect, which is the main subject of discussion herein, they can uniquely cause mitogenic stimulation and enforce quiescent lymphocytes to grow and replicate. Cancer detection and treatment is another field of application of lectins [6–8]. A detailed investigation into the field of lectins would provide valuable information for future antiviral investigations. This review aims to present an in-depth analysis of the most potent antiviral lectins and to shed light on the drug selection path for the clinical use of lectins and drug research and development.

### Structure and classification of lectins

**Fig. 1:**
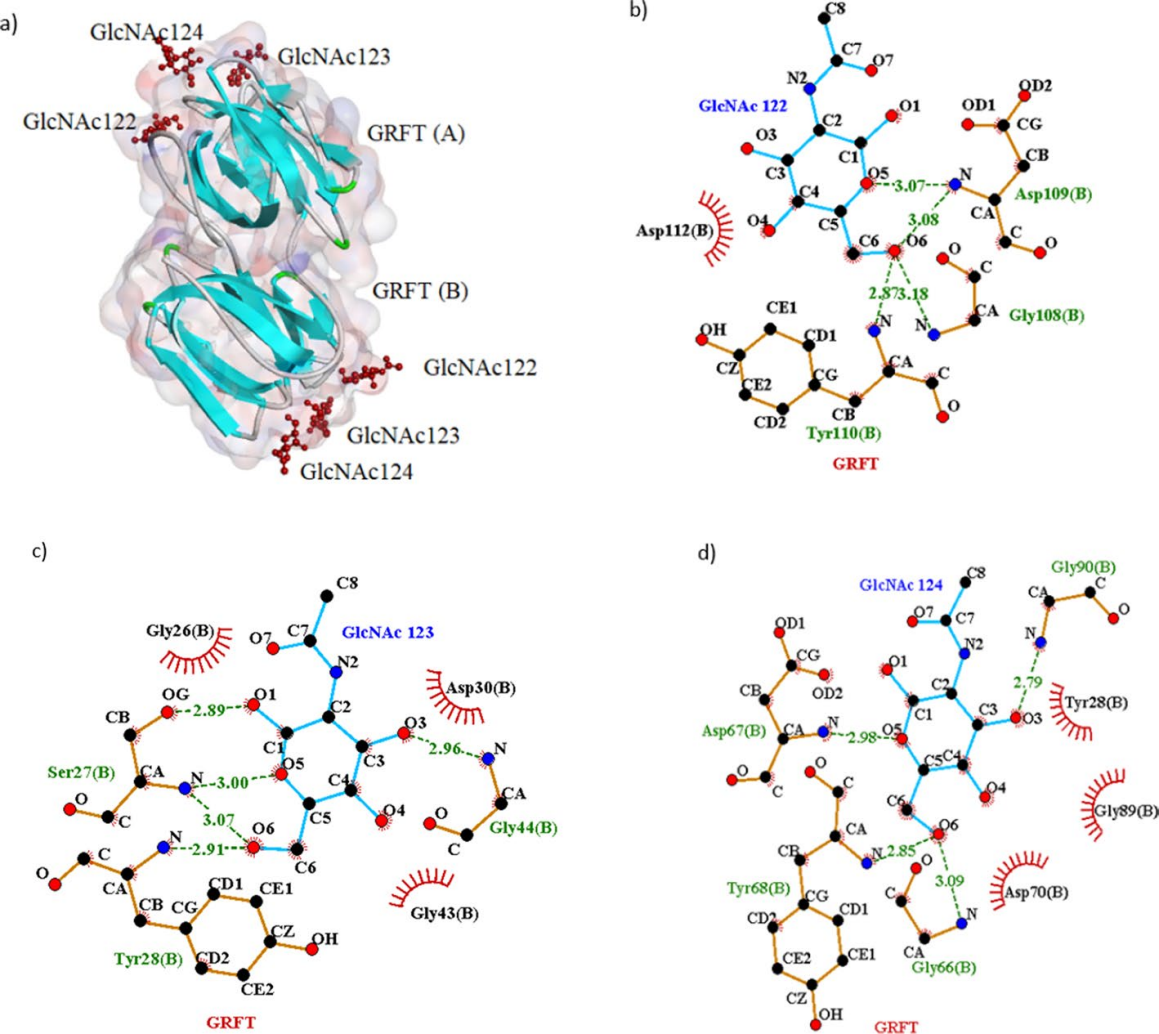
Interaction between lectin griffithsin (GRFT) (PDB ID: 2NU5) that was homodimer (A and B chain) with *N*-acetylglucosamine (GlcNAc) as ligand. **a** The interacting domains of the lectin with GlcNAc, depicted by BIOVIA Discovery Studio Visualizer, are shown. In the figure, the lectin is shown schematically in blue, while ligands are shown in scaled ball-and-stick style in red. **b**–**d** Residues and atoms participating in the interaction between B chain of the lectin and three residues of ligand (GlcNAc 122, 123 and 124), depicted by using the LigPlot^+^ v.2.2 program [15]. The bonds shown by dashed lines in olive-green color represent hydrogenic bonds, while the bonds shown by radius lines in brick-red color represent hydrophobic bonds. The numbers on the hydrogen bond show bond distances. Note: Two carbohydrates of *N*-acetylglucosamine and mannose are contained in SARS-CoV-2 surface glycan, and the residues are exposed to the innate immune system [16]. So, the crystal structure of complexes of antiviral lectin GRFT with glucose and *N*-acetylglucosamine were solved and refined at high resolution. In both complexes, all six monosaccharide-binding sites of GRFT were occupied, and the mode of binding was similar to that of mannose [17]. Therefore, as an example of multiple lectins, the interaction pattern of GRFT with GlcNAc is selected to be shown in the figure

The primary structure of lectins contains carbohydrate-recognition domains (CRDs) with the highest variability, located in their internal repeat sequences. The CRDs are not rigidly fixed in position, such that their structural elements orient them in space, leading to variations in the specific avidity for the three-dimensional (3D) structures of carbohydrates (Fig. 1a–d). In this respect, the sequence similarity of lectins, primarily found in the central region, varies from 10% to 100%. The tertiary structure of different lectins shows great variety, along with the identical function of specific recognition and tight bindings to their ligands. Moreover, some structural elements, namely disulfide bonds and oligomerization, exist in a few lectins but are not prerequisite components. For example, disulfide bonds are located as inter or intradomains of cyanovirin (CV-N) and actinohivin (AH) lectins [9]. In addition, scytovirin (SVN) is a strictly monomeric lectin, consisting of two internal duplicated domains (SD1 and SD2) with 90% identity. These domains are separated by a short linker that is dominantly composed of proline residues. Ten cysteine residues in the SVN constitute five disulfide bridges [10–12]. An example of an intra-disulfide bind is *Serpula vermicularis* lectin (SVL). SVL is a Ca^2+^-independent homotetrameric marine invertebrate lectin (Mw = 12,700), comprising two similar domains connected via disulfide bridges [13, 14].

The classification of lectins is based on the CRD specificity for carbohydrate ligands such as glucose, mannose, *N*-acetylglucosamine, *N*-acetylgalactosamine, and other glycans. Some important antiviral lectins are presented alongside their detailed properties, including origins, structure, and glycan specificity, in Table 1. As mentioned above, the spatial orientation of CRDs in the tertiary structure of lectins enhances their affinity for complex carbohydrates. In line with this finding, several antiviral lectins merely couple with high-mannose oligosaccharides, whereas others possess chitobiose units and high-mannose lateral branches. Duplication of binding domains is another mechanism leading to increased avidity for branched-chain carbohydrates. Besides, binding with linear oligomannose leads to less affinity than the branched structures. Therefore, the spatial position, orientation, and distance of the carbohydrate ligand are essential for the specificity and classification of lectins [9].

**Table 1:** General features of antiviral lectins

Source	Kingdom	Lectin	Mw (× 1000) per monomer	Residue per monomer	Oligomeric status	CRDs	Structure class	Glycan specificity	Refs.
Actinomycete *Longisporum albida*	Bacteria	AH	12.5	114	Monomeric	3	β-Trefoil	α(1,2)-Mannose	[153–156]
Cyanobacterium *Nostoc ellipsosporum*	Bacteria	CV-N	11	101	Monomeric–dimeric	2	Cyanovirin-like	α(1,2)-Mannose	[41, 46]
Cyanobacterium *Microcystis aeruginosa*	Bacteria	MVN	14.2	108	Monomeric	1	Cyanovirin-like	α(1,2)-Mannose	[47–49]
Cyanobacterium *Microcystis viridis*	Bacteria	MVL	13	113	Homodimeric	4	Cyanovirin-like	Man3GlcNAc2, Man6GlcNAc2	[176, 177]
Cyanobacterium *Scytonema varium*	Bacteria	SVN	9.7	95	Monomeric	2	Cyanovirin-like	Man-α(1–2) Man-α(1–6) Man-α(1–6) Man	[10–12]
Cyanobacterium *Oscillatoria agardhii*	Bacteria	OAA	13.9	133	Monomeric	2	OAAH	Man-α(1–6)Man, Man-8/9	[52–55]
*Actinomadura hibisca*	Bacteria	PRM-A	8.5	–	Dimeric	4	–	α(1,2)-Mannose	[58–61]
*Griffithsia* sp.	Protista	GRFT	12.7	121	Homodimeric	6	β-Prism type 1	α(1,2), α(1,6), mannotetrose, man5-9	[103–107, 178]
*Boodlea coacta*	Protista	BCA	13.8	118	Monomeric	3	β-Prism type 1	α(1,2)-Mannose	[64]
*Musa acuminata* cultivars	Plantae	BanLec	15	141	Homotetrameric	8	β-Prism type 1	α-1,6 mannotetrose α-D manno/glycosyl, α-1,3 mannosyl1/β-1,3-glycosyl	[166–170]
*Galanthus nivalis*	Plantae	GNA	12.5	157	Homotetrameric	12	β-Prism type 2	α1-3 or α1-6 linked mannose	[125–129]
*Hippeastrum* hybrid	Plantae	HHA	12.5	157	Homotetrameric	12	β-Prism type 2	α1-3 or α1-6 linked mannose	[125–129]
*Polygonatum cyrtonema* Hau	Plantae	PCL	12	110	Dimeric	6	β-Prism type 2	α(1,3)-Dimannoside	[179–181]
*Urtica dioicia*	Plantae	UDA	8.7	89	Monomeric	2	Hevein-like	(*N*-acetyl-d-glucosamine)_3_	[182–184]
*Nicotiana tabacum* var. *Samsun NN*	Plantae	NICTABA	19	165	Homodimeric	2	Unk	GlcNAc2Man3	[185–187]
*Phaseolus vulgaris*	Plantae	PHA	~ 30	–	Homo/heterotetrameric	4	β-Sandwich	Galβ-(1–4)GlcNAcβ-(1–2)Man	[135–138]
*Lens culinaris*	Plantae	LCA	~ 25	–	Homodimeric	2	β-Sandwich	FucMan3GlcNAc2, Man5-9, GlcNAc	[140–144]
*Serpula vermicularis*	Animalia	SVL	~ 12.7	–	Homotetrameric	–	Unk	*N*-acetyl-d-glucosamine	[13]
*Crenomytilus grayanus*	Animalia	CGL	18	150	Homodimeric	6	β-Trefoil	GalNAc/Gal	[65–73]

*AH* actinohivin, *CV-N* cyanovirin, *MVN* microvirin, *MVL* *Microcystis viridis* lectin, *SVN* scytovirin, *OAA* *Oscillatoria agardhii* agglutinin, *PRM-A* pradimicin A, *GRFT* griffithsin, *BCA* *Boodlea coacta* agglutinin, *BanLec* banana lectin, *GNA* *Galanthus nivalis* agglutinin, *HHA* *Hippeastrum* hybrid agglutinin, *PCL* *Polygonatum cyrtonema* lectin, *UDA* *Urtica dioicia* agglutinin, *NICTABA* *Nicotiana tabacum* agglutinin, *PHA* phytohemagglutinin, *LCA* *Lens culinaris* agglutinin, *SVL* *Serpula vermicularis* lectin, *CGL* *Crenomytilus grayanus* lectin

### Mechanisms of antiviral lectins

Recognition of glycosylated envelope proteins by cell-surface receptors is the usual mechanism for virus recognition and entry. In this respect, lectins coevolved in parallel to impede virus entry and activate host defense mechanisms to neutralize invading viruses. Virus-neutralizing lectins mainly recognize carbohydrate moieties on enveloped viruses in mono- or oligomeric states. They interact with these configurations endowed via CRDs, which are usually repeated in the sequence of lectins, thereby inhibiting viral entry into host cells, on the one hand, and helping the host defense system to find alien viruses on the other hand. Interestingly, duplication of CRDs and multivalent binding features in some lectins are helpful tools by which they can bind to branched sugar moieties on the envelope of viruses more strongly. All of the antiviral lectins characterized and mentioned herein aim to neutralize different viruses by wrapping envelope glycosylated proteins, thereby creating a barrier between these essential recognition tools and their counterpart receptors on the host cell surface [18–22].

### Lectin action on the surface of enveloped viruses

#### Lectins and HIV envelope interactions

**Fig. 2:**
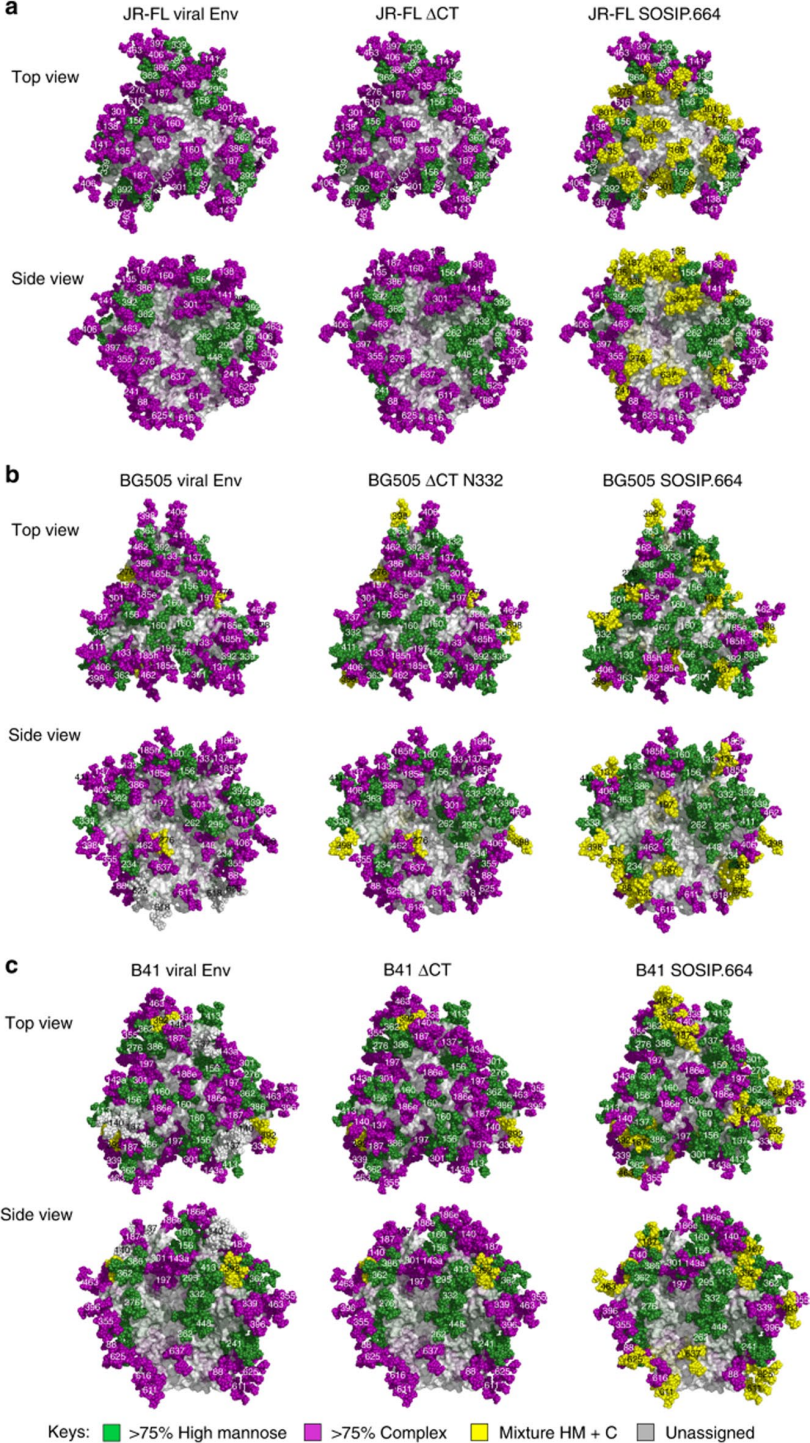
Map of site-specific *N*-glycan processing onto the structure of HIV-1 envelope: **a** JR-FL strain, **b** BG505 strain, and **c** B41 strain. The fully glycosylated models were created with JR-FL ΔCT (PDB: 5FUU), BG505 SOSIP (PDB: 5FYK), and B41 SOSIP. The surfaces of the trimers are represented in grey, and the glycans are represented as spheres colored by the proportion of oligomannose content at that site. The glycans are shown in ball-and-stick representation: > 75% high mannose (green), > 75% complex type glycosylation (purple), mixture of high mannose and complex type glycosylation (25% < high-mannose glycosylation < 75%) (yellow), and the glycosites that were not detected (gray) [25]

As an example of an enveloped virus, the human immunodeficiency virus (HIV) surface is covered with gp120 and gp41. These heavily glycosylated proteins consist of almost 50% glycans. Sugar-binding proteins (such as lectins) specifically and strongly interact with the glycans on the viral surface and disturb the interactions of such invasive viruses with their target receptors on host cells [19, 23, 24]. Mannose oligomers are the central part of HIV carbohydrates that are directed against plant lectins. A map of site-specific *N*-glycan processing onto the structures of the HIV-1 envelope is shown in Fig. 2a–c.

Monocotyledonous families are the best to exemplify HIV-inhibitory plant lectins. Also, reverse transcriptase (RT), the critical enzyme in the life cycle of HIV, is another target of plant lectin. *Pholiota adipose* mushroom, extralong autumn purple bean, and black soybean are the primary sources of plant lectins averting RT activity [26–33]. Cyanovirin-N (CV-N), a lectin that has its origin in blue–green algae, controls a wide range of antiviral properties. Envelope glycoprotein gp120 on the HIV surface is one suggested target for CV-N. CV-N consists of 101 amino acids (Mw = 11,000) with two sequence repeats, exhibiting even specialty toward α-(1–2)-mannose moieties but different affinities for their carbohydrate binding. In this regard, domain B has higher affinity (*K*_d_ = 14 nM) compared with domain A with a lower affinity (*K*_d_ = 1.5 µM) [34–36]. It has been demonstrated that each domain individually does not have sufficient avidity toward gp120 and dissociation constants in the micrometric range, resulting in loss of the virucide function [37–39]. The lectin exists in monomeric and domain-swapped dimer forms, with the latter being unstable at physiological temperatures. However, structure-guided design of CV-N aided the production of a stable domain-swapped dimer form (in a head-to-tail manner), which exhibited elevated anti-HIV activity compared with wild-type CV-N [40].

Multivalency is a common feature of lectins to bind with higher affinity to carbohydrates. In the case of CV-N, the antiviral activity is not enhanced due to geometric constraints when increasing the number of CRDs. Interestingly, Woodrum et al. (2016) reported an increased anti-HIV-virus action (IC_50_ value of 7.5 nM compared with 0.5 nM for WT CV-N) by engineering a flexible dimer of CV-N (called “nested CV-N”), lacking rigidity and spatial constraints [41].

The specific viral targets of CV-N are not limited to HIV and retroviruses. Generally, due to its stable binding, CV-N can inhibit the cytopathic effects of different viruses. For example, CV-N inhibits human influenza A/B strains and the Ebola virus, respectively, by binding to hemagglutinin surface glycoprotein and surface envelope glycoproteins [42–45].

O’Keefe et al. (2003) reported the neutralizing functions of CV-N against viruses at three levels. At level 1, there was no antiviral activity against enteric viruses and rhinoviruses in the first studied category. At level 2, moderate antiviral activity was shown for the second category encompassing herpesviruses and flaviviruses. Finally, at level 3 (which consisted of influenza A and B strains), viruses were susceptible to CV-N except for NWS/33 (H1N1) and A/PR/8/34 (H1N1), which were resistant to even high concentration of CV-N. The results of this study indicate that CV-N hinders viral infection through direct binding and inactivating the invasive virus [43].

Additionally, Maier et al. (2021) designed various domain-swapped dimers differing in the number of B and A domains, then tested their binding affinities against the envelopes of HIV-1, influenza, and Ebola. For the first two, the 2B + 1A form showed higher *K*_d_ values than the other forms including 1B + 1A, 2A, and 1H, suggesting a significant role for multivalency in these bindings. The *K*_d_ values for all the forms against the Ebola envelope protein GP1,2 were similar (*K*_d_ = 26–72 nM) [46].

Another bacterial-derived lectin with high similarity to CVN is a 108-amino-acid lectin with molecular weight of 14,200, specific for Manα-(1–2) Man configurations, named microvirin (MVN). This lectin shows a monodisperse monomer in solution with only one carbohydrate recognition site, very similar to its counterpart in domain A of CV-N [47]. The lectin has slight antiviral potency compared with CV-N, but a narrower antiviral profile (50-fold lower cytotoxicity), which may make it a higher priority for rational engineering to boost its antiviral potency [48, 49]. It can be concluded that the avidity between lectins and mono- and oligosaccharides can be augmented by increasing the number of binding units, thus Yuan-Qin Min et al. (2017) produced oligomer types of MVN, then investigated their binding activities against hepatitis C virus (HCV). All of the oligomers showed higher binding activities compared with the natural monomer type (tetra > tri > di > monomers), while long peptide linkers displayed better anti-HIV binding activity [50]. Munazza Shahid and coworkers (2020) recently engineered an MWN lectin, named LUMS1, by duplicating domain A, which, as a result, is predicted to have higher activity (2CRDs). However, the engineered lectin exhibited less potential against HIV-1 and HCV compared with the unmanipulated lectin (EC_50_ values for HIV-1 and HCV of 32.8–41.6 and 6.6–9.4 nM versus 26.7–63.9 and 31.6–39.5 nM, respectively), apparently indicating the need for further optimization and structures suitable for interaction with carbohydrate moieties [51].

*Oscillatoria agardhii* agglutinin (OAA) is a single polypeptide (133 residues, MW = 13,900) composed of one domain and two CRDs with β-barrel-like topology. The lectin has no affinity for monosaccharides, and its recognition of a pentasaccharide [Man-α-(1–3) Man-α-(1–6) Man-β-(1–4) GlcNAc-β-(1–4) GlcNAc] is pivotal for its binding to Man-α-(1–6) Man disaccharide units [52, 53].

In comparison with all other antiviral lectins, recognizing Man-9 by OAA is unprecedented, while this lectin also has a unique amino acid composition with roughly 20% glycine residues in its amino acid sequence [54, 55]. OAA is a potent anti-HIV-1 lectin with reported EC_50_ values of 30–45 nM [56, 57].

Pradimicin A (PRM-A), an antifungal nonpeptidic benzonaphthacenequinone antibiotic (Mw = 8500), is the first archetype of this class containing d-alanine, d-xylose, 4,6-dideoxy-4-methylamino-d-galactose, and a substituted 5,6-dihydrobenzo[*a*]naphtacenequinone.

PRM-A acts like a c-type “artificial lectin” and predominantly binds to α-(1–2)-mannose configurations. Two molecules of PRM-A cooperate in the carbohydrate-binding processes. First, the binary complex [PRM_2_/Ca^2+^] forms due to the Ca^2+^ bridging effect (i.e., assembly of two molecules of PRM). Next, the ternary complex [PRM_2_/Ca^2+^/Man_2_] organizes by incorporating two molecules of Man with high affinity. Ultimately, the final complex [PRM_2_/Ca^2+^/Man_4_] forms by binding two more Man molecules with low affinity. Thus, PRM-A makes water-insoluble aggregates by cross-linking glycans at the surface of virus particles [58, 59].

PRM-A would be a promising lead for treating various viral infections, particularly HIV. The main advantages of PRM-A include its small size, lack of steric hindrance, that it is unaffected by proteases, chemical stability, neither cytotoxicity nor mitogenicity, quick large-scale production, long-term storage, easy modification, and high genetic barrier [60, 61]. There are numerous binding sites on the gp120 envelope of HIV for PRM-A, following the deletion of some glycans by the virus, i.e., insensitivity to lectin binding. Therefore, not only would the antiviral potential of the lectin not decline but this also makes the virus vulnerable to immune system recognition and elimination. PRM-A inhibits HIV1/2 and simian immunodeficiency viruses (SIVs) with EC_50_ values of 1.6–10 and 5 µM, respectively [62, 63].

*Boodlea coacta* agglutinin (BCA) is a monomeric protein composed of 118 amino acids (Mw = 13,800). Its single domain is separated into three subdomains, whose primary targets are oligosaccharides of α-(1–2)-mannose residues at the non-reducing end (the D1 arm of gp120) with no affinity for internal ones. Studies have shown that influenza H3N2 is the strain that is most affected by BCA (with EC_50_ values of 18.8–74.2 nM). This lectin has also been shown to potently inhibit HIV-1 infection (EC_50_ of 8.2 nM) by binding directly to the gp120 subunit of the virus [64].

Two animal-derived lectins, SVL and CGL, were recently evaluated for their anti-HIV infection properties. *Serpula vermicularis* lectin (SVL) is a Ca^2+^-independent homotetrameric marine invertebrate lectin (Mw = 12,700) comprising two similar domains connected via disulfide bridges. SVL is specific for *N*-acetyl-d-glucosamine monosaccharides and inhibits HIV-1 infection through an unclear mechanism (with an EC_50_ value of 2.8 µM and no cytotoxicity up to > 16 µM) [13, 14]. *Crenomytilus grayanus* lectin (CGL) is a Ca^2+^ independent, dumbbell-shaped, homodimeric protein (Mw = 18,000) whose subunits contain three similar (64–73% identity) tandem subdomains (150 residues per monomer), folded into a β-trefoil configuration. Since the lectin lacks cysteine amino acid, its oligomeric state, presumably, results from hydrophobic interactions [65–70]. CGL has some similarities, such as glycan preferences, with galectins, some of the common animal lectins, but its amino acid sequence is entirely unique. The lectin recognizes ^O^-glycans containing GalNAc/Gal of the nonreducing ends such as mucin-type glycoproteins [71–73]. Regarding recent studies, CGL inhibits the HIV-1 virus (EC_50_ value of 2.5 µM) and has not shown any cytotoxicity (up to a CC_50_ value of 14.6 µM) [14].

#### Lectins as potential molecules in coronavirus treatment/recognition

**Fig. 3:**
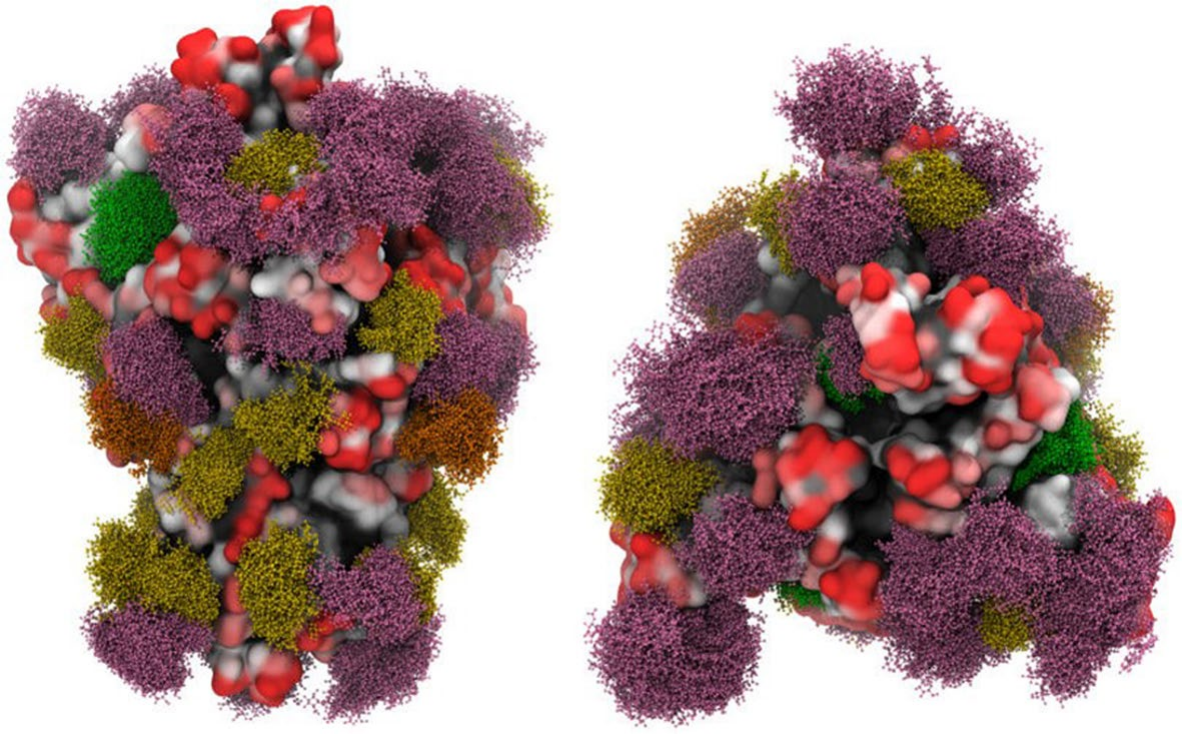
Overlay of snapshots from molecular dynamics (MD) simulation of SARS-CoV-2 S glycoprotein with site-specific glycosylation. The glycans are shown in ball-and-stick representation: high mannose (green), paucimannose (dark yellow), hybrid (orange), and biantennary complex (purple) [82]

Like the mentioned enveloped viruses, severe acute respiratory syndrome coronavirus 2 (SARS-CoV-2) has a glycoprotein envelope around the virion particle. Also, SARS-CoV-2 has glycoproteins on its envelope, suggesting opportunities to use lectins as a treatment strategy [74, 75]. The host cell provides a two-layer envelope for the virus during budding, making its compounds dependent on the cell membrane of origin [76]. Host enzymes glycosylate some of the proteins in the layers of the SARS-CoV-2 envelope. These glycoproteins participate in the adhesion, invasion, and entry of the virus and the formation/modulation of immune system responses. The spike and membrane proteins, called S-protein and M-protein, respectively, are examples of glycoproteins on the envelope of SARS-CoV-2 with important roles in its pathogenesis (Fig. 3) [77–81].

The S-protein arranges as trimers and mediates adhesion between SARS-CoV-2 and the host cell through an interaction with angiotensin-converting enzyme 2 (ACE2) [24, 83–87]. The number of potential glycosylation sites localized in subunits S1 and S2 is 3 for ^O^-glycosylation and 22 for *N*-glycosylation. In this regard, the S1 glycoprotein of SARS-CoV-2 exhibits ligands for several innate immune receptors, particularly C-type lectin receptors (CLRs), which are known to bind specific glycans mainly in a manner dependent on C-type lectin [88]. CLRs, such as macrophage mannose receptor (MMR), macrophage galactose-type lectin (MGL), dendritic cell-specific intercellular adhesion molecule-3-grabbing non-integrin (DC-SIGN), lymph node-specific intercellular adhesion molecule-3-grabbing integrin (L-SIGN), and Dectin-2, are widely expressed by immune system cells such as macrophages, dendritic cells (DCs), and monocytes, which can exert their role as the first line of defense against viruses and pathogens, including SARS-CoV-2 [89].

There is a D614G substitution in the spike protein of all SARS-CoV-2 variants classified as variants of concern (B.1.1.7/alpha, P.1/gamma, B1.351/beta, B.1.617.2/delta, and the newly emerged omicron/B.1.1.529) and variants of Interest (B.1.427/epsilon, B.1.429/epsilon, B.1.525/eta, B.1.526/iota, B.1.617.1/kappa, B.1.617.3, and P2/zeta) called S-D614G, that results in an altered conformation that enhances ACE2 binding and increases transmission and infectivity.

Data show that half of the *N*-glycosylation sequences changed their distribution of glycans in the S-614G variant. The S-D614G variant shows a reduction in the relative level of complex-type glycans (up to 45%) and an increase in oligomannose glycans (up to 33%) in all altered sequences [90]. Han and coworkers [91] also showed different degrees of binding affinity caused by different S glycoprotein mutations, thus indicating that the 501Y.V1 variant yielded the highest enhancements in binding affinity (increased by 36.8%), followed by the N439K variant (increased by 29.5%) and the 501Y.V2 variant (increased by 19.6%). Moreover, N165A and N234A mutations led to glycan deletions at the respective sites, reducing binding of the mutated virus to the ACE2 receptor. This observation suggests that these glycans are actually important for the conformational plasticity of the spike protein receptor binding domain (RBD) of SARS-CoV-2 and hence its ACE2 interaction [92]. Another study investigated the effect of five common SARS-CoV-2 RBD mutations (K417N, K417T, N501Y, E484K, and S477N) on the RBD–ACE2 interaction. They demonstrated that S477N and E484K mutations enhanced transmission primarily by enhancing binding of RBD and ACE2, while K417N and K417T mutations decreased this affinity. It was also indicated that N501Y, E484K, K417N and K417T mutations facilitated immune escape [93]. So, mutations in spike protein S1 of SAR-CoV-2 improve its affinity with ACE2 and, consequently, increase its infectivity and pathogenicity.

On the other hand, through the Janus-activated kinase (JAK)/signal transducer and activator of transcription (STAT) signaling pathway, interferon-stimulated genes (ISGs) are activated by binding interferins to their receptors on the cell surface, leading to immune responses [94]. In this regard, ACE2 is one of the ISGs, and its expression levels correlate with type I interferons. Reduced levels of ACE2 in the lung are beneficial for the host in controlling viral replication and transmission. Nevertheless, if there is insufficient ACE2 for a prolonged period of time, angiotensin II would not be properly converted into ang1-7 by the function of ACE2. The resulting accumulation of angiotensin II will have a negative impact on immune activation and may eventually cause lung disease. After binding of SAR-CoV-2 spike protein S1 to ACE2, it downregulates ACE2 and type I interferons, which may directly contribute to SARS-CoV-2-associated lung disease [95]. It has also been indicated that induction of ACE2 and type I interferons by poly I:C, an interferon inducer, is suppressed by S1 protein in primary cells of lung bronchoalveolar lavage (BAL) from naive rhesus macaques. Thus, the S1 and S2 glycoproteins are golden targets for the host immune system and drug and vaccine design, including the lectibodies discussed in the next section. In this regard, glycosylated nonstructural proteins (e.g., the 3a protein, which plays a pivotal role in SARS-CoV-2 virulence) are also suitable targets for lectibodies [78, 96–102].

In this regard, griffithsin (GRFT) is a potent antiviral lectin (121 amino acids, Mw = 12,700) with domain-swapped dimer folding. High-resolution crystallographic experiments have revealed three identical carbohydrate-binding sites per monomer, resembling a β-prism-1 motif. Each CRD recognizes one terminal mannose monosaccharide on *N*-linked Man5-9GlcNAc2 configurations. Indeed, GRFT shows antiviral activity against a broad range of viral infections, including HIV (EC_50_ values of 0.03–1.3 nM), HCV (6.7–13.9 nM), and SARS-CoV (48 nM) [103–107].

This property is attributed to its favorable preclinical features such as lack of toxicity and mitogenicity, synergistic effect with various other lectins and drugs, and inexpensive bulk production [108, 109]. In this regard, GRFT has been the subject of two phase I clinical studies investigating its toxicity in healthy populations. One part of a two-part study looked at the safety of GRFT injected intravaginally for a single dose, followed by 14 consecutive days of use in healthy women. The second part of the study involved 30 subjects receiving placebo and 30 subjects receiving GRFT gel. The results of cell-based assays and cervical explants showed that GRFT was safe for vaginal use up to 14 days with potent anti-HIV activity [110, 111].

Another phase I clinical study on GRFT (PREVENT, pre-exposure prevention of viral entry) has been conducted since 2014. GRFT was studied for the purpose of providing a comprehensive dataset that would facilitate an informed decision on whether the topical microbicide should proceed. Study students who were HIV-1 seronegative and engaged in unprotected receptive anal interactions (URAI) in this double-blind, randomized, phase 1 study were given GRFT enema rectally [112].

However, a recent preformulation study on GRFT by Kramzer et al. (2021) revealed that slow oxidation of GRFT occurs following long-term storage (at methionine 78, in particular). Hence, more studies are required to protect GRFT from oxidation [113–123]. GRFT also shows synergy when used together with other antivirals. Cai et al. (2021) reported that the combination of EK1 (which binds to the HR1 in the S2 subunit of SARS-CoV-2) and GRFT (which binds to the RBD in the S1 subunit of the same virus) potently inhibits the SARS-CoV-2 virus. In this respect, the EC_50_ values of GRFT and EK1 alone were 511 and 2459 nM, respectively, while the EC_50_ for GRFT-L25-EK1 (where L25 refers to the 25-mer linker) was 20 nM, demonstrating the efficacy of the synergism [124].

*Galanthus nivalis* agglutinin (GNA) and *Hippeastrum* hybrid agglutinin (HHA) are two lectins that highly resemble each other, in terms of their monomeric molecular weight (12,500), oligomeric status (homotetrameric, resulting from noncovalent interactions among four monomers), number of sugar-binding sites per monomer (three CRDs), and secondary structure (β-prism-type 2). Nevertheless, they show distinct potential binding targets; while GNA exclusively binds to α-(1,3)-mannose ends, HHA CBAs can corecognize internal and external α-(1,3)-or α-(1,6)-linked mannose moieties. In addition to binding to mannose termini, GNA also recognizes some other carbohydrates, including lactosamine structures found on *N*- and/or *O*-glycans [125–127]. These lectins have dual antiviral activity: First, by their numerous carbohydrate-recognition sites, they bind to several target sugars, then force the target virus to eliminate some glycans on its envelope. Second, now that mutant virus strains expose their protein motifs to the immune system, Nab can now simply trigger immune responses against the invading virus [128, 129]. Both GNA and HHA have demonstrated potent antiviral activities against SARS-CoV (6.2 and 3.2 µg/ml, respectively) [130–134].

Moreover, phytohemagglutinin (PHA) shows affinity toward *N*-glycans of complex type, but not monosaccharides. PHA is considered to be a “complex-type” specific lectin, recognizing the Gal-β-(1–4)GlcNAc-β-(1–2)Man structure [135]. There are two types of subunits, named E (because of binding to erythrocytes and then agglutinin activity) and L (owing to its association with leukocytes and then mitogenic activity), that assemble into five different types of tetramers (Mw = ~ 120,000), i.e., E4, E3L1, E2L2, E1L3, and L4 [136, 137]. Despite the 70% sequence similarity between the E and L subunits, they have different glycan-binding preferences. Indeed, while PHA-E prefers terminal Gal and GlcNAc glycans, PHA-L recognizes Man residues through interaction with both α1–3 and α1–6 branches [138].

Wang et al. (2021) recently investigated the antiviral potency of PHA-E and L against SARS-CoV-2 pseudovirus and reported no cytotoxicity with EC_50_ values of 141–200.1 and 184.9–217.9 nM, respectively [139].

*Lens culinaris* agglutinin (LCA) is a homodimeric lectin in solution, where each monomer is composed of two distinct polypeptides, viz. α (the light chain with 52 amino acids) and β (the heavy chain with 180 residues), which assemble to an α2β2 composition (with molecular mass of 49,000) with a β-sandwich structure [140–142]. Binding to one metal ion (usually Mn^2+^) and one Ca*2+* per subunit is obligatory for sugar binding (one CRD per monomer) [143, 144]. Lentil lectin needs a FucMan3GlcNAc2 core for its binding; it can bind to *N*-glycan oligosaccharides of Man-5 to Man-9 with the highest affinity, as well as GlcNAc residues at the nonreducing terminals with lower affinity. LCA has been shown to increase HIV infection and transmission to CD4^+^ cells through an unknown mechanism, likely due to the high expression level of fucosylated glycans on the surface of eukaryotic cells [145]. More recently, Wang and colleagues (2021) showed for the first time that LCA has potent antiviral activity against SARS-CoV-2 pseudovirus (with EC_50_ values of 152.3–186.6 nM) with no cytotoxicity effect [139]. A list of antiviral lectins with their cytotoxicity and mitogenicity properties and effective concentrations used to treat enveloped viruses is presented in Table 2.

**Table 2:** Toxicity and antiviral activity of lectins

Lectin	Cytotoxicity	Mitogenicity	Activity (nM unless otherwise noted)	Refs.
AH	No	No	HIV-1 (2–110), HIV-2 (3–14),	[148, 157–164]
CV-N	Yes	Yes	HIV (0.1–33.7), HCV (1.6), Ebo (100)	[34–40]
MVN	No	No	HIV-1 (2–167), HCV (31–39)	[50, 51]
MVL	Yes	Unk	HIV-1 (30–37), HCV (14–34)	[188, 189]
SVN	No	Unk	HIV-1 (0.3–22), EBOV (41)	[190–194]
OAA	Yes	Unk	HIV-1 (30–45)	[56, 57]
PRM-A	No	No	HIV-1/2 (1.6-10 µM), SIV (5 µM)	[62, 63]
GRFT	No	No	HIV (0.03–1.3), HCV (6.7–13.9), SARS-Cov (48), JEV (20), HSV-2(230), HPV (0.4–1.39 µM), NiV (20–60), ANDV (180–230)	[104–109, 113–124, 178, 195, 196]
BCA	No	Unk	HIV-1 (8.2), influenza H3N2 (18.8–74.2)	[64]
BanLec	Yes	Yes	HIV-1 (0.8–14), HIV-2 (3.7)	[171–174]
GNA	No	Unk	HCV (11.1–25.5), influenza A H1N1 (0.1–268), influenza A H3N2 (0.4–6.4), influenza B (0.016–0.89), HIV-1 (0.3–4.7 μg/ml), HIV-2 (0.1–0.2 μg/ml), SIV (2.7 μg/ml), FIV (0.09 μg/ml), SARS-COV (6.2 μg/ml), FIPV (3.9 μg/ml)	[130–134]
HHA	No	Unk	Influenza A H1N1 (0.05–121), influenza A H3N2 (0.10–3), influenza B (0.015–1.8), HIV-1 (0.3–3.2 μg/ml), HIV-2 (0.1–0.2 μg/ml), SIV (0.6 μg/ml), FIV (0.1 μg/ml), SARS-COV (3.2 μg/ml), FIPV (2.6 μg/ml)	[130–134]
PCL	No	Unk	HIV-1 (0.05–0.08 μg/ml), HIV-2 (0.08–0.1 μg/ml)	[179, 197]
UDA	Yes	Yes	HIV-1(100–180), HIV-2 (240–420), HSV-1(9.6- > 11 µM), HSV-2 (1.1–1.3 µM), SIV (130–190), MSV (> 20 µg/ml), SARS-CoV (0.9–2.6 µg/ml), influenza A H1N1 (5–435), influenza A H3N2 (5.8–83), and influenza B (0.64–14)	[182–187, 198–203]
NICTABA	No	Unk	HSV-1 (171–263), HSV-2 (41–64), influenza A H1N1 (21–43), influenza A H3N2 (13–23), influenza B (11), RSV (105), and DENV type 2 (323–729)	[107, 176–178]
PHA	No	Yes	SARS-CoV-2 (141–217.9), HIV-1 (50 µg/ml)	[116, 122]
LCA	No	Unk	SARS-CoV-2 (152.3–186.6)	[116]
SVL	No	Unk	HIV-1 (2.8 µM)	[14]
CGL	No	Yes	HIV-1 (2.5 µM)	[14]

*HIV* human immunodeficiency virus, *HCV* hepatitis C virus, *EBOV* Ebola virus, *SIV* simian immunodeficiency virus, *SARS-CoV* severe acute respiratory syndrome coronavirus, *JEV* Japanese encephalitis virus, *HSV* herpes simplex virus, *HPV* human papillomavirus, *NiV* Nipah virus, *ANDV* Andes orthohantavirus, *FIV* feline immunodeficiency virus, *FIPV* feline infectious peritonitis virus, *MSV* maize streak virus, *RSV* respiratory syncytial virus, *DENV* dengue virus

### New generation of lectins: lectibodies

Besides their size limitations, short stability in the body environment, vulnerability to proteolytic lysis, and challenges regarding bulk production, the cytotoxicity, mitogenicity, and proinflammatory properties of lectins raise several questions regarding their valuable usage as antiviral agents. In this regard, several protein engineering techniques have been applied to produce modified lectins, such as lectibodies, to overcome these issues [146, 147].

**Fig. 4:**
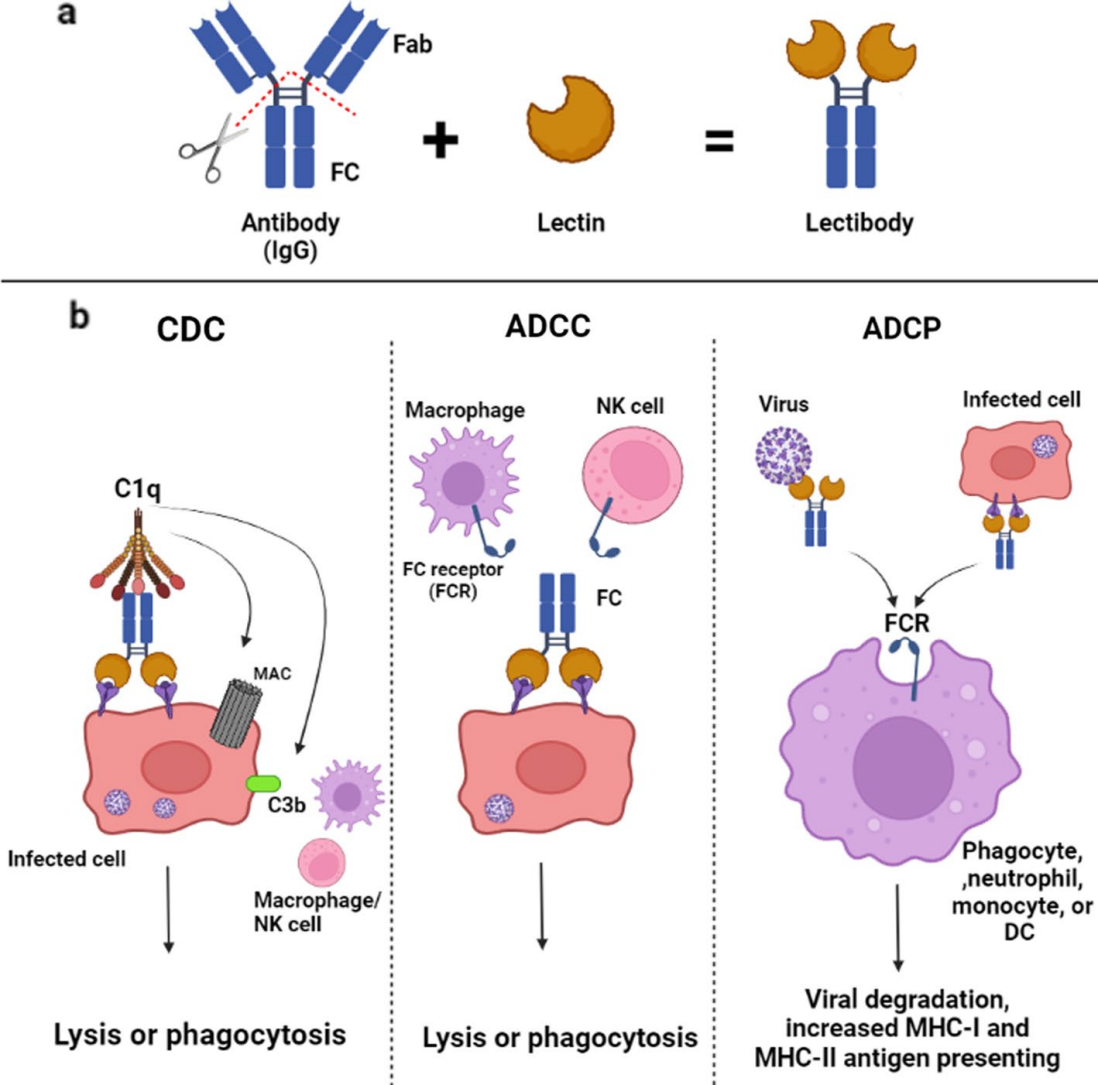
**a** Fusion of lectin and antibody’s crystallizable fragment (Fc) of immunoglobulin G (IgG) produces a molecule called a “lectibody.” **b** Lectibodies can bind to surface glycoproteins via their lectins, neutralize viruses or cells infected by viruses, and help the innate and adaptive immune systems function against pathogens through functions including complement-dependent cytotoxicity (CDC), antibody-dependent cell-mediated cytotoxicity (ADCC), and antibody-dependent cell-mediated phagocytosis (ADCP)

Fusion of lectin and antibody’s crystallizable fragment (Fc) of immunoglobulin G (IgG) produces a molecule called a “lectibody” that can act as a carbohydrate-targeting antibody (Fig. 4a). Lectibodies can bind to surface glycoproteins via their lectins, neutralize viruses or cells infected by viruses, and perform Fc-mediated antibody effector functions that include complement-dependent cytotoxicity (CDC), antibody-dependent cell-mediated cytotoxicity (ADCC), and antibody-dependent cell-mediated phagocytosis (ADCP) (Fig. 4b) [148].

The CDC mechanism is activated by binding of C1q to Fc-bound virus-infected cells, initiating the cascade. By releasing C3 and C5 molecules, immune effector cells are recruited and activated, while C3b binds to pathogens and infected cells to initiate immune complex clearance and phagocytosis. Following assembly of a membrane attack complex, infected cells undergo lysis.

The ADCC response is triggered by the binding of Fc gamma receptor (FcγR) on natural killer (NK) cells to Fc domain on antibodies bound to viral antigens on infected cells [149]. Upon release of cytotoxic granules, infected cells are killed. Liu et al. demonstrated that anti-Ebola monoclonal antibodies have predominantly NK cell ADCC activity [150].

During ADCP, phagocytic cells ingest virus–antibody or antibody–infected cell complexes. The antigen is then processed and presented on major histocompatibility complex (MHC) molecules on cell surfaces, or transferred to lysosomes to be degraded. ADCP has been shown to reduce SARS-CoV infection when anti-SARS-CoV antibodies were administered to mice [151, 152].

CVN-Fc, as a lectibody, has an intense inhibiting activity on free enveloped viruses. CVN-Fc prevents virus attachment and entrance into the target infection cell and attracts host defense cells to the virus war zone. HCV, HIV, Ebola, and influenza are among the list of CVN-Fc’s target viruses. In addition, actinovirin (AH), an actinomycete-derived lectin, is a single polypeptide with 114 residues (Mw = 12,500) that folds into a β-trefoil structure. This structure has three highly conserved tandem repeats (HMG-binding pocket) specific toward α-1,2-mannose oligomers (D1) of high-mannose-type glycans (HMTGs) on the HIV envelope [153–156]. Chiba et al. (2004) reported the inhibitory activity of AH against T- and M-tropic HIV-1 strains (with IC_50_ values of 2–110 nM and 38 nM, respectively) and HIV-2 (with an IC_50_ value of 3–14 nM). They also found that, although three CRDs in AH recognize gp120 on the env-expressing cells specifically and cooperatively, they have no significant affinity toward chemokine receptor-expressing cells such as CD4^+^ [157]. Besides the antiviral properties of AH, it is highly hydrophobic and prone to aggregate. Therefore, Hamorsky et al. (2019) produced a soluble AH variant in *Nicotiana*, with more efficient biochemical and pharmaceutical properties, named avaren (AV). They also fused AV to the fragment Fc fragment of IgG1 to create the avaren-Fc (AvFc) lectibody. Avaren-Fc (AvFc) is a binding neutralizing lectibody for HIV and simian immunodeficiency virus (SIV) strains, without affecting normal human blood cells, which attaches to the HMGs on the gp120 of HIV envelope. AvFc exhibited an extended serum half-life in rats and macaques, whereas repeated systemic administration in mice did not result in any noticeable toxicity. Moreover, surface plasmon resonance (SPR) analysis revealed that AvFc has tenfold higher affinity to the gp120 of HIV relative to AH, indicating the high selectivity and specificity of the lectibodies. Currently, no pharmaceutical agent chooses HMG selectively as a target. Therefore, developing AvFc and/or lectibodies in general may be unprecedented for selectively rendering HIV and SIV ineffective subtypes [158].

HCV is another enveloped virus that is densely glycosylated with HMGs. In addition to facilitating cell entry through surface receptors, these HMGs act as an armor-like shield in front of neutralizing antibodies. Previous studies have shown the affinity of AvFc to a recombinant HCV E2 envelope glycoprotein and its ability to prevent viral infection of Huh-7-hepatocyte-derived cellular carcinoma cell by cell culture-derived HCV (HCVcc).

Although the antiviral function of AH is not the strongest when compared with other lectins, it does not show any cytotoxicity and mitogenicity, which are common side effects among lectins. Takahashi et al. (2011) demonstrated that, via dimerizing AH proteins, its activities against various HIV strains can be increased due to the “cluster effect” of lectin (with IC_50_ values of 12–290 nM) [159–161]. Hydrophobic nature and high aggregation rate are the major drawbacks of AH. Hence, developing a more efficient recombinant product is necessary to make lectin a potent anti-HIV drug. Its soluble variant Avaren was obtained by structure-guided mutations and fused to Fc of human IgG1. As a result, the “lectibody” AvFc was created, which is more active against HIV infection (with IC_50_ values of 5.6 and 0.3 nM for HIV-1 and 2, respectively) compared with AH (with IC_50_ values of 60.4 and 156.3 nM against HIV-1 and 2, respectively). It was also reported that AvFc might inhibit SIV (with IC_50_ values of 3.8–15.3 nM) and HCV (with IC_50_ values of 1.69–2.85 nM) [148, 158, 162–164]. These results suggest that lectibodies can act as inhibitors of viral entry into host cells and represent a potential solution to reduce the mortality rate of hepatitis C [165].

Banana lectin (BanLec) is a 141-residue plant-derived lectin (Mw = 15,000). Native mass spectrometry (MS) demonstrated that BanLec assembles as a homotetramer in solution, with each subunit folding into a β-prism-1 topology, characteristic of Jacalin-related lectins (JLRs). There are two sugar-binding sites in each subunit (octavalent for native tetrameric BanLec), recognizing 1,3-sugar moieties, particularly α/β-d-mannosyl/glycosyl configurations at the reducing ends, plus internal α-1,3-linked glucosyl residues. Concomitantly, the lectin recognizes numerous *N*-glycans on different HIV trimers, making the virus particles cross-link and aggregate [166–170].

The major drawback of BanLec is its mitogenic effect on T-cells. However, it has been shown previously that an engineered type of this lectin (BanLec H84T) avoids any mitogenicity while retaining the broad-spectrum antiviral functions. The rationally engineered lectin inhibits HIV-1 and HIV-2 with EC_50_ values of 0.4–4.1 nM and 0.3 nM, whereas the EC_50_ values for the wild type are 0.8–14 nM and 3.7 nM, respectively [171–173]. Moreover, a fluorescently labeled BanLec (BanLec-eGFP) was produced recently by Lopandic et al. (2021) and exploited as a versatile detection tool to investigate tissues and pathological processes structurally and functionally [174].

In recent years, IgG monoclonal antibodies have played an essential role in producing therapeutic drugs (> 70 antibody drugs) for many diseases, such as cancer, autoimmune illnesses, and viral infections. The Fc domain in IgG is constituted from two constant domains CH2 and CH3, which are responsible for the ADCC and complement activation produced by IgG. Previously, lectibodies were produced by fusing a lectin and Fc domain genetically. However, such production of full-length antibodies in large quantities in bacterial systems is challenging because disulfide bonds needed for proper folding cannot be created. Moreover, large-scale production of antibodies in mammalian expression systems is expensive and slow, and also needs more time for optimization, in addition to the problem of heterogeneous expression of foreign proteins from different organisms. More recently, Jaakkonen et al. (2020) exploited an off-the-shelf approach as a solution for the production of Fc fusions, where each domain of a bivalent lectibody preproduced in their ideal host organism and expression system is then ligated to produce engineered bispecific antibodies. This approach avoids the time-consuming optimization of the expression and purification steps, while only the ligation step needs to be optimized. Indeed, the heavy-chain antibodies in camelids, which bear only two heavy chains, inspired this novel approach. They used in vitro protein *trans*-splicing (PTS) to replace the antigen-binding domains of IgG with the SVN lectin to produce a lectin fusion protein, i.e., lectibody, that can specifically attach to the HMGs on the surface of diverse virus particles such as HIV, SARS coronavirus, and Ebola virus, on the one hand, and call the immune system to wipe out these viruses out, on the other. They also documented that the size of the Fc domain and its separate valency could, respectively, enhance the stability and binding capacity of the small protein SVN [175].

## Conclusions and perspectives

Lectins are potent antiviral agents with a promising future for the treatment/recognition of viral infections. Unlike most antiviral compounds that inhibit virus replication, antiviral lectins target the entry of viruses into cells, leading to lower toxicity in topical use. However, since various obstacles prevent their clinical use, future investigations should focus on these. The risks or limitations to the significant use of antiviral lectins are their size, short stability in the body environment, cytotoxicity and mitogenicity (for some lectins), the potential for awakening the immune system (thereby resulting in deleterious responses), vulnerability to proteolytic lysis, and challenges regarding affordable bulk production. Fortunately, computational tools or gene manipulation can produce more effective derivatives of these valuable proteins, such as lectibodies, to improve their stability and robustness and remove their mitogenic potential. In this regard, lectibodies can be considered to represent a new and promising approach, with high specificity and cost-effectiveness, to treat viral infections by identifying and neutralizing carbohydrates on the surface of the viral envelope. Lectibodies can not only neutralize the virus and inhibit its entry into the host cell through specific cell surface receptors but also, with the assistance of immune system mechanisms such as CDC, ADCC, and ADCP, induce the clearance of the virus or infected cells. Virus envelope glycans may be able to exert their role as a shield to reduce the normalizing function of antibodies against the virus. However, lectibodies may be superior in this respect, especially in terms of the performance of vaccines against newer variants of a virus that is consistently developing new mutations. However, because lectibodies have recently provided new insight into viruses, particularly SARS-CoV-2 and HIV, further studies and accurate clinical trials are needed to investigate their tissue distribution and effect on tissue metabolism and confirm their specificity and engagement of cell receptors, to prevent their possible binding to unwanted glycosylated targets or undesirable local immune responses.

## Data Availability

Not applicable.

## Abbreviations

HMGs: High proportion of high-mannose-type glycans
PRM-A: Pradimicin A
CRDs: Carbohydrate recognition domains
CV-N: Cyanovirin
AH: Actinohivin
SVN: Scytovirin
SVL: Vermicularis
GRFT: Griffithsin
GlcNAc: *N*-acetylglucosamine
HIV: Human immunodeficiency virus
RT: Reverse transcriptase
MVN: Microvirin
HCV: Hepatitis C virus
OAA: *Oscillatoria agardhii* agglutinin
SIV: Simian immunodeficiency virus
BCA: *Boodlea coacta* agglutinin
CGL: *Crenomytilus grayanus* lectin
SARS-CoV-2: Severe acute respiratory syndrome coronavirus 2
ACE2: Angiotensin-converting enzyme 2
CLRs: C-type lectin receptors
MMR: Macrophage mannose receptor
MGL: Macrophage galactose-type lectin
DC-SIGN: Dendritic cell-specific intercellular adhesion molecule-3-grabbing non-integrin
L-SIGN: Lymph node-specific intercellular adhesion molecule-3-grabbing integrin
DCs: Dendritic cells
GNA: *Galanthus nivalis* agglutinin
HHA: *Hippeastrum* hybrid agglutinin
PHA: Phytohemagglutinin
LCA: *Lens culinaris* agglutinin
Fc: Crystallizable fragment
IgG: Immunoglobulin G
CDC: Complement-dependent cytotoxicity
ADCC: Antibody-dependent cell-mediated cytotoxicity
ADCP: Antibody-dependent cell-mediated phagocytosis
FcγR: Fc gamma receptor
NK: Natural killer
MHC: Major histocompatibility complex
SPR: Surface plasmon resonance
HCVcc: Cell culture-derived HCV
BanLec: Banana lectin
JLRs: Jacalin-related lectins
PTS: Protein *trans*-splicing
BanLec: Banana lectin
CRDs: Carbohydrate recognition domains
